# Contour-Based Corner Detection and Classification by Using Mean Projection Transform

**DOI:** 10.3390/s140304126

**Published:** 2014-02-28

**Authors:** Seyed Mostafa Mousavi Kahaki, Md Jan Nordin, Amir Hossein Ashtari

**Affiliations:** Center for Artificial Intelligence Technology, Faculty of Information Science and Technology, Universiti Kebangsaan Malaysia (UKM), Bangi, Selangor 43600, Malaysia; E-Mails: jan@ftsm.ukm.my (M.J.N.); amirhossein@ftsm.ukm.my (A.H.A.)

**Keywords:** corner detection, contour-based corner detector, mean projection transform, polygonal approximation

## Abstract

Image corner detection is a fundamental task in computer vision. Many applications require reliable detectors to accurately detect corner points, commonly achieved by using image contour information. The curvature definition is sensitive to local variation and edge aliasing, and available smoothing methods are not sufficient to address these problems properly. Hence, we propose Mean Projection Transform (MPT) as a corner classifier and parabolic fit approximation to form a robust detector. The first step is to extract corner candidates using MPT based on the integral properties of the local contours in both the horizontal and vertical directions. Then, an approximation of the parabolic fit is calculated to localize the candidate corner points. The proposed method presents fewer false-positive (FP) and false-negative (FN) points compared with recent standard corner detection techniques, especially in comparison with curvature scale space (CSS) methods. Moreover, a new evaluation metric, called accuracy of repeatability (AR), is introduced. AR combines repeatability and the localization error (*L_e_*) for finding the probability of correct detection in the target image. The output results exhibit better repeatability, localization, and AR for the detected points compared with the criteria in original and transformed images.

## 1. Introduction

Feature detection is a fundamental issue in image processing and computer vision that is directly related to interest points. Corner points are considered important features for feature extraction [1]. Corner detection is a low-level image processing technique that is widely used in different computer vision applications [2], such as camera calibration [3], target tracking [4], transformed image identification (TII) [5], image registration [6], 3D polyhedral building modelling from aerial imagery [7], multi-scale feature extraction from LIDAR data [8], 2D and 3D building extraction [9,10], and automotive applications [11]. However, different approaches require a different perspective for the corner definition. Historically, the terms of the corner point refer to the terms of both the interest point and the region of interest [12]. Generally, the corner detection in an image is the point on the contour at which two straight edges meet at a particular angle or the location at which the direction of the contour changes significantly [2].

Numerous corner detection methods have been introduced over the last several decades. These methods can be divided into three main categories: intensity-based detectors [13–17], model-based detectors [18,19], and contour-based detectors [1,4,20–25]. Each category has its own competencies for different types of areas and images. Recently, the third category has received more attention in terms of robustness and efficient computational cost. Model-based detectors extract the corner points by matching a predefined corner model to the image and calculating the similarity for detecting corner points. Their algorithms limit the detection to specific tasks, such as finding chessboard corners [3]. For general and flexible corner detection, defining a general corner model is difficult and does not cover all types of corners for different image types with different scene properties. Intensity-based detectors attract more attention than model-based detectors [1]. Intensity-based detectors use the grey-level information of the image to detect the corner points by applying the first- or second-order derivative on the images. The second-order derivatives of intensity-based methods are noise sensitive and are rarely used in the literature [1]. In 1977, Moravec [14] introduced the idea of finding the corner points as ‘points of interest’ that have high-intensity variations in the vertical and horizontal directions. Harris and Stephen [15] proposed the most famous corner detector method, known as the Harris (Plessey) method, to improve upon Moravec's idea. The Harris method is based on approximates of the auto-correlation of the gradient in different directions. The Harris method is the most well-known method in the literature, but it cannot detect high-order corners [1]. A high-order corner is a point at which three or more contour regions meet [1]. The Harris method uses the Gaussian filter to reduce the FP corners in noisy images and increases the localization accuracy of the detector. Noble [26] proved that the Harris corner detector is only robust in ‘L’-type corners. Based on these weaknesses, Shi and Tomasi [13] improved the Harris detector with a minor correction and calculated the minimum eigenvalues. Smith and Brady [16] introduced the Smallest Uni-value Segment with an Assimilating Nucleus (SUSAN), which used a gradient convolution of a circle mask called the USAN area to detect the corner points on a grey-level image. Yang *et al.* [27] improved the SUSAN method using a self-adoptive threshold and a rotating coordinate system, but the method was not sufficient for high accuracy of localization. Several improvements have been proposed for the Harris and SUSAN methods [28–36]. Grey-scale methods are sensitive to noise and are not as accurate for detecting the exact corner point location.

Robustness to noise is an important issue for contour-based detectors [37], and researchers have proposed several algorithms over the last decade to address this problem. Contour-based detectors consist of three main steps: edge detection, contour extraction, and decision making on the contour [1]. The basic idea of contour-based methods was proposed by Rosenfeld and Johnston [23] in 1973 to calculate the angle of the curves on digital imagery. Subsequently, Kitchen and Rosenfeld [38] introduced their corner detector based on the change in direction of the gradient (first- and second-order derivatives) on the contour. This method is considered the first cornerness measure of the edge map in the literature. Coeurjolly *et al.* [39] extended the Worring and Smeulders [40] corner classification to a discrete method based on an estimation of the discrete osculating circle. Nguyen and Debled-Rennesson [41] extended the estimator proposed in [39] using blurred segments. Malgouyres *et al.* [42] introduced a discrete binomial convolution for a convergent estimator to reduce the noise effect. Kerautret and Lachaud [43] subsequently introduced a discrete curvature estimation-based method to calculate the curvature radius passing from the corner points.

Over the last two decades, curvature scale space (CSS) methods have been widely used as corner detectors in the literature due to their high performance. CSS-based detectors exhibit some weaknesses, which are considered in this paper. They generally use second-order derivatives, which can cause an increase in the FP rate because of contour variation. Additionally, they require a Gaussian scale selection to smooth the curve area, which is application based and a difficult task. The basic idea was introduced by Rattarangsi and Chin [44] in 1992, and the basic CSS-based methods were proposed by Mokhtarian and Suomela [20] in 1998 and modified by Han and Poston [21] in 2001. CSS-based detectors use several planar curves that are smoothed using multi-scale Gaussian functions to calculate the local curvatures. Thresholding is used to remove the FP corner points from the candidate corners. CSS-based detectors are sensitive to noise on the contour, and the curvature estimation uses high-order derivatives to reduce the localization accuracy and high false rate [37]. A large-scale Gaussian function reduces noise but affects the corner localization, whereas a small-scale Gaussian function is sensitive to noise. To address these problems, Awrangjeb and Lu [37] proposed chord-to-point distance accumulation (CPDA) using the adoptive threshold method based on Han and Poston's idea [21]. The CPDA method uses a discrete curvature estimation that is more robust to the local variation. These authors used three chords of different lengths to estimate three normalized discrete curvature values at each point of the smoothed curve. They then multiplied the normalized values to achieve the curvature product. The candidate corners were selected from the maximum of the absolute curvature products. Because intensity variation information is not effective for extracting the corner candidate [1], a universal corner model (UCM) was proposed in [1] using the anisotropic directional derivative (ANDD) filter to improve the CPDA method to reduce the effect of the intensity variation of the contour and improve the localization accuracy. The proposed kernel in the ANDD filter is a Gaussian-based kernel based on sampling the continuous anisotropic functions with ρ as the anisotropic factor and σ as the scale parameter. Because the ANDD method is based on an anisotropic Gaussian kernel for smoothing, it changes the contour to the curve, and it is difficult to select an appropriate Gaussian scale [45]. Thus, ANDD is insufficient for detecting corners with both a high detection rate and repeatability with an acceptable *L_e_*. Elias and Laganiere [25] proposed a method named JUDOCA, which defined the junctions as a meeting point of two or more ridges in the gradient domain. The region of a circle mask to measure the cornerness is used after edge detection and Gaussian filtering to detect the corners. An edge extraction process in CSS-based detectors is a sensitive operation that may cause the original corner point in the contour to be missed and the diagonal lines to be aliased on the edge. Anti-aliasing filters cannot affect the edge map. These problems affect the FP rate and localization accuracy of the detectors. Some studies combine corner detection categories to achieve better performance. Escalera and Armingol [3] used a hybrid corner detection to extract corners on a chessboard using the Hough transform for the contour and then established the chessboard corner models, but this method is limited to a specific task.

In this paper, a new projection transform, called mean projection transform (MPT), is proposed to extract the corner candidates and address the aliasing problem. Next, a parabolic fit approximation is used to determine the corner points in the extracted candidates. This method reduces problems related to the existing CSS-based algorithms. The proposed method is compared to the detectors presented in [1,25,37] because these detectors claim to provide better detection performance compared to the other available methods.

This paper is organized as follows: Section 2 discusses the MPT method for selecting corner candidates. Section 3 presents the parabolic fit approximation to confirm corner points from the MPT candidates and localize them. Section 4 discusses the evaluation results and proposes a new corner detection evaluation method called AR, which addresses the limitations of the current evaluation metrics for FP and FN points; the proposed corner detector is then assessed using *L_e_*, repeatability, and AR.

## 2. Mean Projection Transform

A new projection transform based on the mean of integral values in both the horizontal and vertical directions is proposed. Contour-based detectors use contour information to extract the corner candidates and corner points. Based on CSS problems regarding contour aliasing and variation, the MPT method is proposed to extract the corner candidates. MPT representation guarantees that the detector only selects candidates that have high curvature, and it addresses the aforementioned problems.

### 2.1. Global Mean Projection Transform

MPT is a transform that consists of the integrals over straight lines in a digital image. If *f*(*X*) = *f*(*x, y*) is a function of the image signal (L) in ℝ^2^, then MPT is a transform of L, where the mean of the integrals in vertical and horizontal directions is calculated using Equation (1):

(1) $$\text{MPT}(\text{L})=\text{Mean}\left(\int_{\text{LX}}f(\text{X})\left|\text{dx}\right|,\int_{\text{Ly}}f(\text{X})\left|\text{dy}\right|\right)$$

The arc-length *t* on the line (L) can be written as Equation (2):

(2) $$\begin{array}{cc} \left(\text{x}(\text{t}),\text{y}(\text{t})\right) & \\ & =\frac{1}{2}[\left((\text{t}\;\sin(\alpha )+\text{s}\;\cos(\alpha )),(-\text{t}\;\cos\left(\alpha \right)+\text{s}\;\sin\left(\alpha )\right)\right)\\ & +((-\text{t}\;\cos\left(\alpha \right)+\text{s}\;\sin\left(\alpha \right)),\text{t}\;\sin\left(\alpha \right)+\text{s}\;\cos\left(\alpha \right)))] \end{array}$$

where s is the Euclidean distance from the origin to L, α is the angle of the vector, and L is in the Cartesian coordinate system. (α, s)are the transform parameters on ℝ^2^ for all lines, and MPT can be represented in the aforementioned coordinates according to Equation (3):

(3) $$\text{MPT}=(\alpha ,\text{s})=\frac{1}{2}\int_{-\infty }^{\infty }f\left(\text{x}(\text{t}),\text{y}(\text{t})\right)\text{dt}+\int_{-\infty }^{\infty }f\left(\text{y}(\text{t}),\text{x}(\text{t})\right)\text{dt}$$

This equation can also be written as:

(4) $$\begin{array}{cc} \left(\text{MPT}(\alpha ,\text{s})\right) & \\ & =\frac{1}{2}(\int_{-\infty }^{\infty }f\left(\left(\text{t}\;\text{sin}(\alpha )+\text{s}\;\cos(\alpha )\right),\left(-\text{t}\;\cos\left(\alpha \right)+\text{t}\;\sin\left(\alpha \right)\right)\right)\text{dt}\\ & +\int_{-\infty }^{\infty }f\left(\left(-\text{t}\;\cos\left(\alpha \right)+\text{t}\;\sin\left(\alpha \right)\right),\left(\text{t}\;\sin\left(\alpha \right)+\text{s}\;\cos\left(\alpha \right)\right)\right)\text{dt}) \end{array}$$

The MPT that considers the multi-directional integral can be formulated as Equation (5):

(5) $$\text{MPT}(\text{ρ},\text{τ})\left[f(\text{x},\text{y})\right]=\frac{1}{2}(\int_{-\infty }^{\infty }f({\text{x},\text{τ}+\text{ρ}}_{\text{x}})\text{dx}+\int_{-\infty }^{\infty }f({\text{y},\text{τ}+\text{ρ}}_{\text{y}})dy)$$

where ρ is the slope of line L, and τ is the intercept factor.

**Figure 1.**
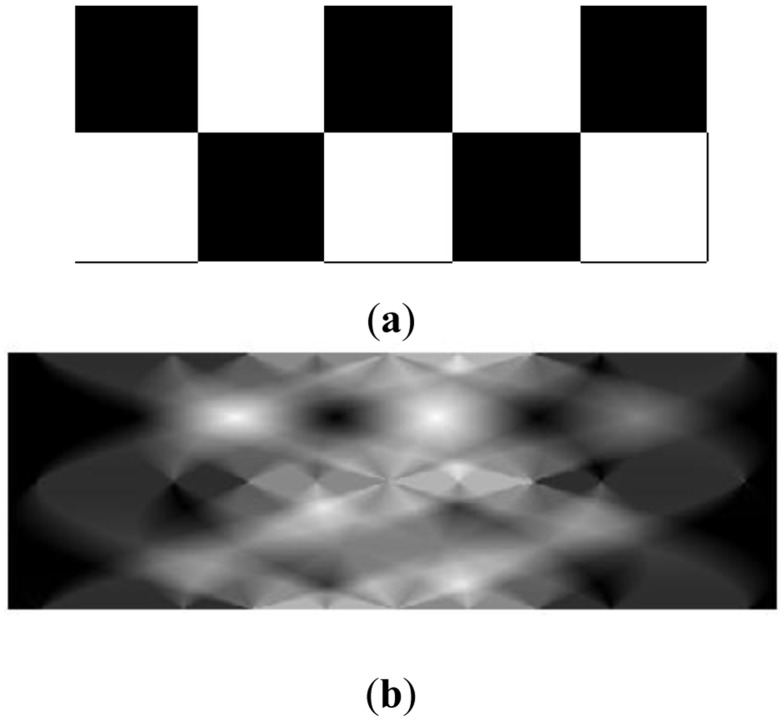
Chessboard image as (**a**) original image; (**b**) MPT result.

MPT calculates the mean of the integrals in an input image in both the vertical and horizontal directions of line L. The parameters of MPT can detect the available angular contours from a straight contour on the edge map of the objects in an image. The MPT of the sample image is shown in Figure 1. The image contains at least a corner where the MPT representation of the image includes more than a segment or peak.

MPT calculates the integral of *f*(*x*, *y*) for each line and the mean of the vertical and horizontal integrals in all directions θ є [0,2π). The output of MPT has more than a peak for each significant change in the contour direction. The coordinate of a corner point may not be extracted using the MPT function, but the corner candidates can be extracted to address the aliasing problem of CSS-based detectors and reduce the FP rate significantly.

### 2.2. Projection of the Corner

**Figure 2.**
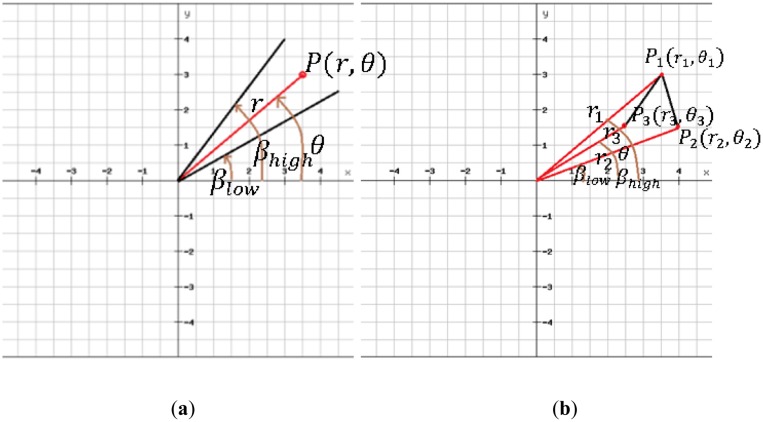
(**a**) Illustration diagram of the BCM [1]; (**b**) Diagram of the PUCM.

A projection of universal corner model (PUCM) describes all corner types. The basic corner model (BCM) of a curve is the area in which the horizontal and vertical integrals are significantly different than the non-corner model (NCM). In the polar coordinate system, the BCM and NCM can be defined by Equation (6) [1]:

(6) $$\text{BCM}(\text{r},\text{θ})=\{\begin{array}{rr} 1, & 0\leq \text{r}\leq +\infty ,{\text{β}}_{\text{low}}\leq \text{θ}\leq {\text{β}}_{\text{high}}{,\text{β}}_{\text{high}}-{\text{β}}_{\text{low}}\neq \text{π}\\ 0, & \text{otherwise} \end{array}$$

where *r* is the radial coordinate, and θ is the polar angular coordinate *β*_low_ is the lower band, and *β*_high_ is the upper band of θ. In Figure 2, the BCM and PUCM are presented graphically. Considering the integral projection, PUCM can be defined by:

(7) $$\text{PUCM}(\text{R},\text{θ})=\{\begin{array}{rr} 1, & 0\leq \text{r}\leq +\infty {,\text{r}}_{3}\leq {\text{r}}_{2}\leq {\text{r}}_{1}=>{\text{θ}}_{2}\leq {\text{θ}}_{3}\leq {\text{θ}}_{1}\\ 1, & 0\leq \text{r}\leq +\infty {,\text{r}}_{3}\leq {\text{r}}_{2}\leq {\text{r}}_{1}=>{\text{θ}}_{1}\leq {\text{θ}}_{3}\leq {\text{θ}}_{2}\\ 0, & \text{otherwise} \end{array}$$

where θ = [θ _1_, θ _2_, θ _3_] denotes the polar angles, and R = [r_1_, r_2_, r_3_] is the radius of three points of the curve. Figure 2a presents the BCM introduced by [1], and a new universal projection of the corner model is defined in Figure 2b. Based on Equation (7), different types of corner shapes can be described in polar coordinates.P_3_ is assumed as the middle point in the polar coordinate system in terms of θ. The value r in the same coordinate system follows Equation (7) to satisfy the corner properties. The PUCM representation of a corner point is the mean integral projection of the local values of the image. This process employs the MPT of the BCM to obtain the analytic expression of the PUCM projection representation. The projection of the input can identify whether there is angular contour.

The MPT representation of the BCM is presented in Equation (8):

(8) $$\begin{array}{cc} \partial \nabla_{\beta_{\text{low}},\beta_{\text{high}}}^{\text{BCM}} & =\iint_{{\text{R}}^{2}}{\text{BCM}}_{\beta_{\text{low}},\beta_{\text{high}}}(\text{r},\text{θ}){\text{ψ}}_{\text{σ},\text{ρ},\text{θ}}(-\text{r},-\text{θ})\text{rdrdθ}\\ & =\frac{1}{2\text{π}}\{\frac{\cos(\text{θ}-{\text{β}}_{\text{low}})}{\sqrt{\cos^{2}\left(\text{θ}-{\text{β}}_{\text{low}}\right)+{\text{ρ}}^{4}\sin^{2}(\text{θ}-{\text{β}}_{\text{low}})}}\\ & +\frac{\sin(\text{θ}-{\text{β}}_{\text{low}})}{\sqrt{\sin^{2}\left(\text{θ}-{\text{β}}_{\text{low}}\right)-{\text{ρ}}^{4}\cos^{2}(\text{θ}-{\text{β}}_{\text{low}})}} \end{array}$$

**Figure 3.**
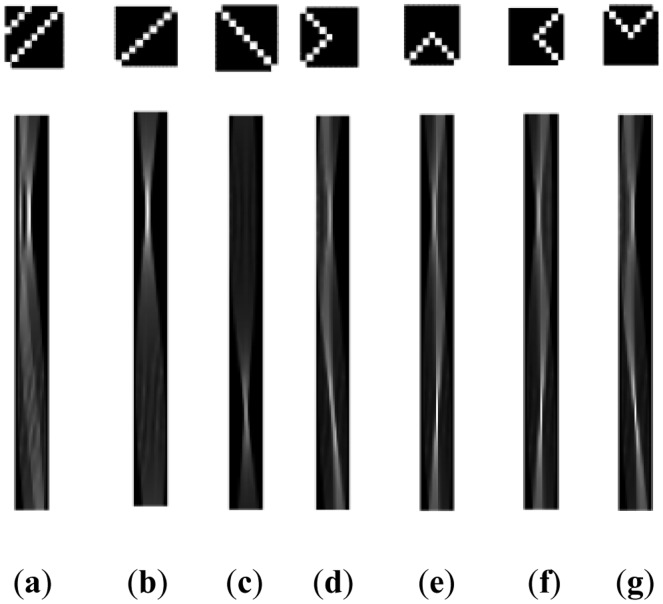
Contour models: (**a**) NCM of double line model (top) with its MPT representation (bottom); (**b**) Diagonal NCM (top) and its MPT representation (bottom); (**c**) Diagonal NCM (top) and its MPT representation (bottom); (**d**) UCM (top) and its PUCM representation (bottom); (**e**) UCM (top) and its PUCM representation (bottom); (**f**) UCM (top) and its PUCM representation (bottom); (**g**) UCM (top) and its PUCM representation (bottom).

Equation (8) has two zero values for θ = β ± π and two extremes for θ = β and θ = β + π. The results for different directions are the projection of the object for both the vertical and horizontal views simultaneously. Some corner models and non-corner models are manually extracted from the contour of the object, and their MPT representations are shown in Figure 3.

**Figure 4.**
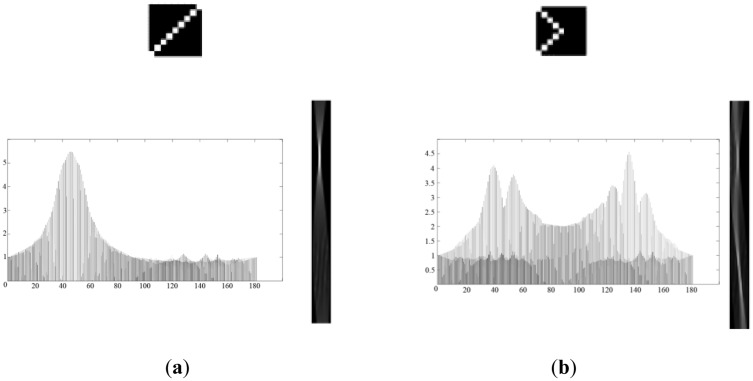
Different MPT representation peaks in (**a**) NCM (top), its MPT representation (bottom-right) and MPT plot (bottom-left); (**b**). PUCM (top), its MPT representation (bottom-right) and MPT plot (bottom-left).

As shown in Figure 4b, the PUCM of the corner models has at least two separated peaks in the MPT representation because the integral values in the horizontal and vertical directions are calculated. In contrast, the straight line has only one peak in the MPT model, as Figure 4a demonstrates. The input image is swept by a moving window to select all candidates using MPT. The default moving window is 9 × 9, but the size of the moving window is an initial parameter that can be adjusted based on the image size. In large-scale images, the moving window size should be large enough to detect corners properly.

## 3. Corner Point Detection: Approximation of the Parabolic Fit

**Figure 5.**
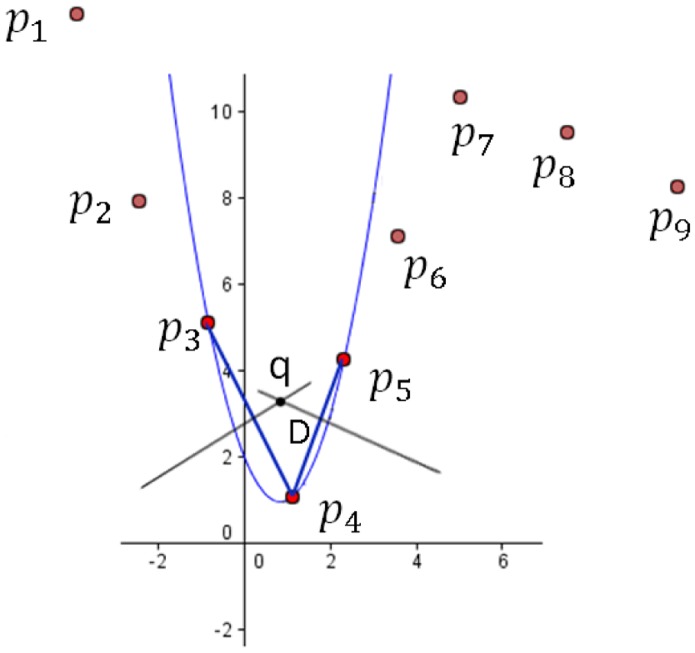
Approximation of the parabolic fit estimation technique.

Curvature extraction and angle estimation are the key features of the contour-based corner detection methods. CSS detectors extract the curve, analyze the curvature properties of the contour map, and then detect the corner points. Γ is considered the curvature at a point, as presented in Equation (9) [44]:

(9) $$\Gamma =\frac{\text{dψ}}{\text{ds}}$$

where ψ is the change rate of angle, and the corresponding S can be defined as the arc-length. Curve smoothing reduces sensitivity to the local variation of the contour [20]. The CSS-based detectors, which use contour smoothing, are not sufficient to detect the corner points based on their evaluation results. However, selecting a general σ value for smoothing is a difficult task and can affect the localization performance of the detectors. To address these problems, a multi-scale curvature estimator using parabolic fit approximation to detect the corner points is presented. P = 〈p_1_, p_2_, …., p_n_〉is the n points on the curve Γ(t) = (x(t), y(t)) with a given distance function d(p_i_, p_j_), ρ(p, r) = {q|(p, q) ≤ r} is a parabola with the radius *r*, and center q and p_i_ are the points inside the area, as shown in Figure 5.

Orthogonal lines meet at the center point of the parabola ρ, which are defined as D. If ρ_i_ denotes ρ (p_i_, ε), then p_i_p_j_ is a segment δ_h_(p_i_p_j_) ≤ ε if p_i_p_j_ intersect at d_i+1_,…,d_j−1_, and the parabola radius passing the points is:

(10) $$\text{r}=\frac{{\left[1+{\left(\frac{\text{dy}}{\text{dx}}\right)}^{2}\right]}^{\overset{3}{\;}/\underset{2}{\;}}}{\left|\frac{{\text{d}}^{2}\text{y}}{{\text{dx}}^{2}}\right|},$$

where *dx* and *dy* are extracted using p_i_ to P_j_ points inside the parabola area. Additionally, the proposed method is adjustable for detecting the low- and high-order corners in different image scaling by adjusting the values of ϑ as the focal control parameter. The general definition for ε is:

(11) $$ɛ>\frac{w\times 2}{\vartheta },$$

where ϑ is the focal control parameter, and *w* is the moving window width. The truth condition in Equation (11) guarantees that the ε value does not exceed the curve radius. ϑ and *w* are input arguments that are adjustable by the user to support high scaling images. ϑ and *w* are 5 and 9 by default, respectively, for a 512 × 512 image.

Generally, the approximation of the parabolic fit is robust to the local variation [46]. Therefore, it can estimate the curvature without the curve-smoothing process. Compared to other CSS-based estimators, the proposed method is not sensitive to the aliasing of the edge map; thus, it detects the corner points with higher performance.

## 4. Experimental Results and Evaluation Metrics

**Figure 6.**
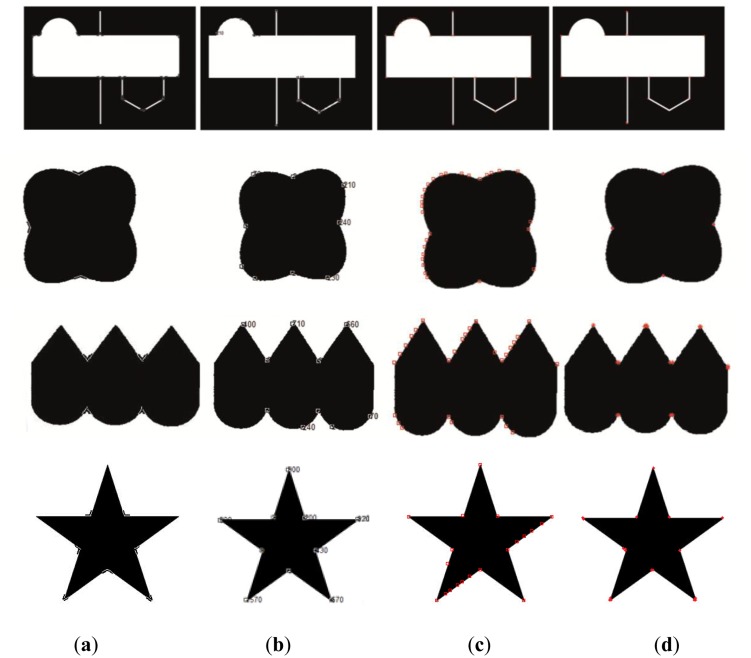
Results of the different corner detection techniques. (**a**) JUDOCA; (**b**) CPDA; (**c**) ANDD; (**d**) Proposed method.

To evaluate the proposed method, a dataset called “*image database and corner detection*” [47] and some standard images that are commonly used in the corner detection assessments are applied. The compared criteria are CPDA [37], ANDD [1], and JUDOCA [25], which claim to have the most accuracy among the current standard methods. The repeatability, *L_e_*, and AR are employed as the performance comparison metrics. All dataset images are transformed with different types of attacks for use as test inputs. Eighteen different rotated images have angle θ in [−90°, +90°] at 10° apart, excluding 0°. The combined transformations, including rotation and scale transform with different rotations θ in [−20, +20] at 10° apart and scale factors s_x_, s_y_ in [0.9,1.3], are used for assessment. Figure 6 presents some sample results of the different methods in a normal image situation. Some FP corners are detected due to an aliasing issue on contours, especially in the ANDD method, as shown in Figure 6c.

**Figure 7.**
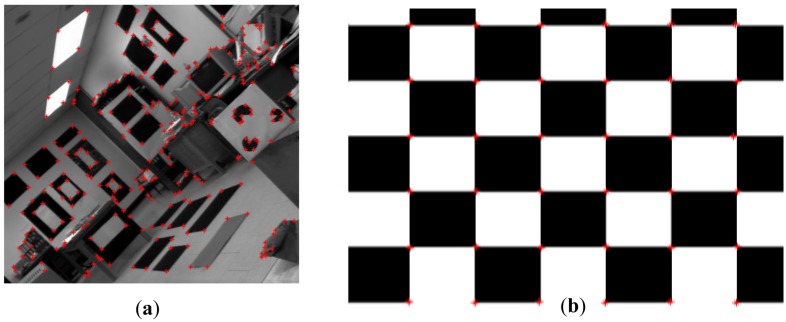
Corner detection results (**a**) Lab and (**b**) Checkerboard.

In addition to the simple images, the proposed method indicates good performance in complex shapes. Figure 7 shows two commonly used grayscale images, a 512 × 512 Lab and 1,600 × 1,163 checkerboard used in the experiment as samples. Accurate chessboard corner detection is quite useful for camera calibrations, as used in [3].

### 4.1. Receiver Operating Characteristic (ROC)

**Figure 8.**
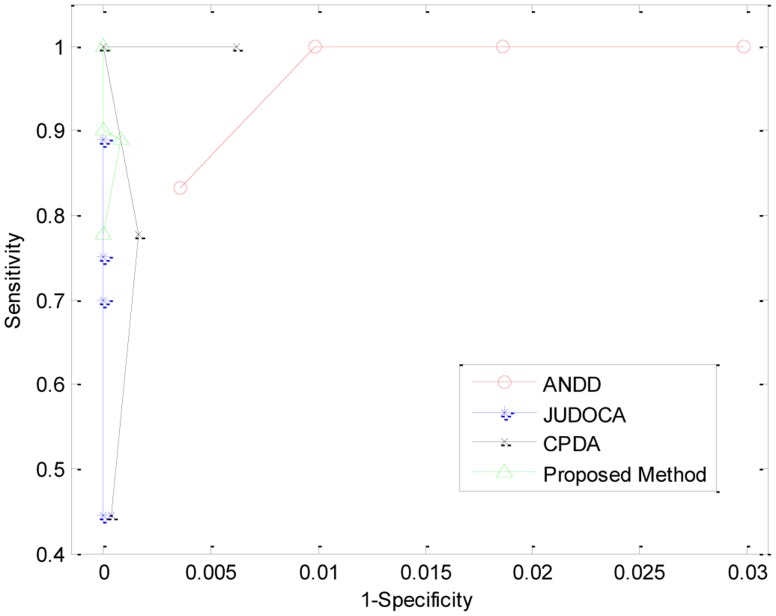
ROC plot comparison of the proposed method, ANDD, JUDOCA, and CPDA.

In detection theory, the receiver operating characteristic, or ROC, is a graphical plot that illustrates the performance of the system based on detection rates to provide a more appropriate comparison [48]. In this section, we used the ROC to compare the performance of different methods based on FP and true-positive (TP) rates to calculate sensitivity and specificity. Specificity relates to the detector's ability to identify negative results. Sensitivity is the ability of a detector to identify positive results. Higher sensitivity shows few FNs, and low specificity shows many FPs. Figure 8 illustrates the ROC plot of four detectors.

When comparing the performance of the detectors considering the ROC plot, a detector is better when its plot points are located on the top-left side of the plot area, which shows higher sensitivity and specificity. To determine the FNs and FPs, human judges generate the ground truth [49].

Among the detectors, CPDA attains comparable detection performance with the proposed method. The proposed method concentrates in the top-left of the graph, which indicates higher TPs and few FPs, indicating higher performance. JUDOCA provides the lowest FPs in comparison with the others, whereas ANDD shows many FPs and fewer FNs.

### 4.2. Localization Error

**Figure 9.**
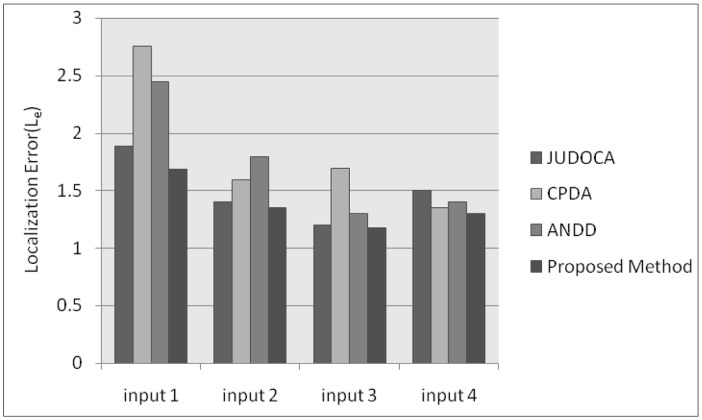
Comparative results of the different methods under *L_e_*.

**Figure 10.**
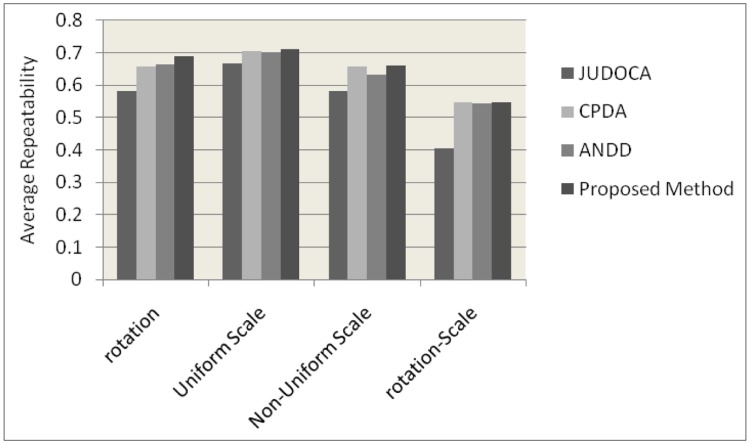
Average repeatability under rotation, uniform scale change, non-uniform scale change, and the combined rotation and scale effect of the different methods.

**Figure 11.**
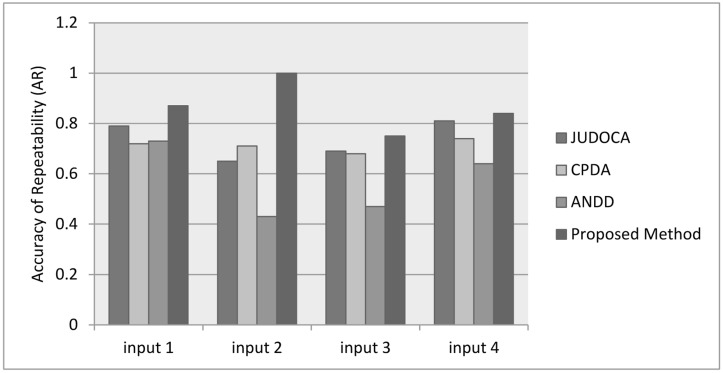
Comparative results of the different methods under the *AR*.

The *L_e_* is a common evaluation method for a corner detector [50]. *L_e_* can measure the robustness and accuracy of the detected corners and can be defined by:

(12) $${\text{L}}_{\text{e}}=\sqrt{\frac{1}{{\text{N}}_{\text{r}}}\sum_{\text{i}=1}^{{\text{N}}_{\text{r}}}\left[{\left({\text{x}}_{\text{oi}}-{\text{x}}_{\text{ti}}\right)}^{2}+{\left({\text{y}}_{\text{oi}}-{\text{y}}_{\text{ti}}\right)}^{2}\right],}$$

where x_oi_ and y_oi_ are the ground truth coordinates of the corners, x_ti_ and y_ti_ are the coordinates of the *i*-th detected corner, and N_r_ is the total detected points of the detector. Figure 9 presents the comparative results of the different methods under *L_e_*. Four types of input are selected to calculate the error. On average for all inputs, the proposed method indicates the best *L_e_* result, followed in descending order by JUDOCA, ANDD, and CPDA. *L_e_* is not a reliable evaluation metric to rank the detectors, as we discuss in Section 4.4. According to Figures 10 and 11, CPDA provides better results than ANDD and JUDOCA, but the *L_e_* result for CPDA is lower than the others because *L_e_* does not consider FPs and FNs directly. *L_e_* only considers the detected points (TPs) and their locations in the target image.

### 4.3. Average Repeatability

Average repeatability (*R_avg_*) is another evaluation method in the literature related to the corner and interest point detectors [1,51]. This method is more reliable than *L_e_* to show the robustness of the detector because it automatically calculates the average number of detected corners in the original and transformed images. This method is easier to implement, can be completely automatic without human operations and is more secure in terms of human mistakes. Average repeatability measures the robustness of the detector for different transformations and can be defined by:

(13) $${\text{R}}_{\text{avg}}=\frac{{\text{N}}_{\text{r}}}{2}\left(\frac{1}{{\text{N}}_{\text{o}}}+\frac{1}{{\text{N}}_{\text{t}}}\right),$$

where N_o_ is the number of corners in the ground truth, and N_t_ is the number of detected corners. N_r_ is the repeated corners between two results within a maximum three-pixel error rate. Figure 10 presents the comparison results of repeatability for different detectors and image attacks.

To compare the proposed method with other methods, the same dataset with the same conditions is used to evaluate the results. The proposed method outperforms the other methods in different conditions. For the combined rotation and scale transform, after the proposed method, the best results are achieved by CPDA [37]. The worst average repeatability for all of the methods is for the combined rotation and scale transforms. The proposed method indicates higher average repeatability results for different effects compared to the other methods. This average repeatability is achieved because the powerful MPT method for candidate selection is applied to detect the initial candidates from the original image. Therefore, the aliasing problem, which causes several FP detections, is addressed, and the approximation of the parabolic fit method supports the localization performance by finding the point coordinates of the corners.

### 4.4. Accuracy of Repeatability

When comparing the effect of the transformation on the results, the repeatability and localization parameters are not sufficient because they do not directly consider FPs and FNs. FPs and FNs are quite important in corner detection methods and should directly affect the evolution result. Moreover, average repeatability does not consider the ground truth information, which means that it does not determine whether the detected points in the original image are localized correctly. Therefore, a new comparison method based on both the *L_e_* and the repeatability is proposed. The new comparison method, known as AR, is sensitive to FNs and FPs and thus significantly affects the FNs and FPs given in the results. Therefore, AR is a good measurement technique for corner detection methods.

In the proposed comparison method, each corner point is analyzed to provide a probability of P_i_, and the mean of probability for all points generates the AR, as defined in:

(14) $$AR=\frac{1}{N}\sum_{i=1}^{N}P_{i},$$

where N is the largest number of corner points of either the result image from the corner detector or the ground truth image. Let us assume that the ground truth corner points are G_j_, and the result points are R_i_. Each R_i_ has a corresponding P_i_ ∈ [0,1]. The value ‘1’ exhibits the highest probability that is a TP, and the value ‘0’ exhibits the lowest probability of the corner point that is either an FN or FP. The number of points in the ground truth and result image is M and M′ respectively. For the two points in the ground truth and result image, the distance is calculated by:

(15) $${\text{d}}_{\text{ij}}=\sqrt{{\left({\text{x}}_{\text{oj}}-{\text{x}}_{\text{tj}}\right)}^{2}+{\left({\text{y}}_{\text{oi}}-{\text{y}}_{\text{ti}}\right)}^{2}}$$

where x_oj_ and y_oj_ are the ground truth corner coordinates, and x_ti_ and y_ti_ are the coordinates of the *i*-th detected corner of the detector. A matrix D is M × M′ as shown in Equation (16). It is defined to save the Euclidian distances between the corresponding points in the ground truth and result images:

(16) $$\text{D}=\left\lceil \begin{array}{ccc} d_{11} & \cdots & d_{1M^{\prime }}\\ \vdots & \ddots & \vdots \\ d_{M1} & \cdots & d_{MM^{\prime }} \end{array}\right\rceil$$

In the first step, δ_xy_ = min(D) = d_xy_ is obtained. Then, column y and row x corresponding to d_xy_ are eliminated. Therefore, matrix D is M−1 × M′−1. This process continues until all elements in matrix D are eliminated. In each step, δ_xy_ = min(D) = d_xy_ is the closest distance between the ground truth G_x_ and the result point R_y_. For each point, the probability is calculated using the maximum size of the ground truth or result image, as defined in Equation (17). The maximum distance in an image is its diagonal, which is the maximum error in localization. Thus, dividing by the maximum error gives a normalized P*_ij_* between 0 and 1 that can be assumed as the correctness probability of the TP location:

(17) $$P_{ij}=1-\frac{\delta_{ij}}{\sqrt{\max{\left(m,m^{\prime }\right)}^{2}+\max{\left(n,n^{\prime }\right)}^{2}}}$$

where the size of the ground truth is m × n, and the size of the result image is m′ × n′. The result of *AR* on the four input images is shown in Figure 11. In all inputs, the proposed method indicates better performance. Among the detectors, ANDD shows the worst AR in most of the inputs because of its greater FP detected corners. JUDOCA and CPDA indicate approximately the same result in AR comparison and have the second best result after the proposed method.

## 5. Conclusions and Future Work

This paper introduced a new corner detection method based on contour information. Candidate selection using a new image transformation called MPT was the basic approach of this paper. MPT calculates the mean of the integral of the image contour in both the horizontal and vertical directions. After selecting the corner candidates by MPT, an efficient curvature estimation based on parabolic approximation was used to confirm and localize the corner points in the candidates. The results were evaluated by *L_e_* repeatability, and AR, which indicate the robustness and accuracy of the proposed method. AR was proposed as an evaluation metric that highlights FP and FN more than other metrics in the assessment results. The proposed method outperforms the other standard methods in terms of *L_e_*, repeatability, and *AR*. Future work may research the projection of the corners in different aspects and may result in better corner candidate selection and a higher repeatability and AR. An efficient corner detection algorithm can be used in different computer vision applications, such as point matching, mobile robot vision, and image registration.
